# Mutation Analysis of *MYORG* in a Chinese Cohort With Primary Familial Brain Calcification

**DOI:** 10.3389/fgene.2021.732389

**Published:** 2021-10-18

**Authors:** Yi-Heng Zeng, Bi-Wei Lin, Hui-Zhen Su, Xin-Xin Guo, Yun-Lu Li, Lu-Lu Lai, Wan-Jin Chen, Miao Zhao, Xiang-Ping Yao

**Affiliations:** Department of Neurology and Institute of Neurology of First Affiliated Hospital, Institute of Neuroscience, and Fujian Key Laboratory of Molecular Neurology, Fujian Medical University, Fuzhou, China

**Keywords:** primary familial brain calcification, MYORG, mutations, parkinsonism, phenotype

## Abstract

Primary familial brain calcification (PFBC) is a progressive neurological disorder manifesting as bilateral brain calcifications in CT scan with symptoms as parkinsonism, dystonia, ataxia, psychiatric symptoms, etc. Recently, pathogenic variants in *MYORG* have been linked to autosomal recessive PFBC. This study aims to elucidate the mutational and clinical spectrum of *MYORG* mutations in a large cohort of Chinese PFBC patients with possible autosomal recessive or absent family history. Mutational analyses of *MYORG* were performed by Sanger sequencing in a cohort of 245 PFBC patients including 21 subjects from 10 families compatible with a possibly autosomal-recessive trait and 224 apparently sporadic cases. In-depth phenotyping and neuroimaging features were investigated in all patients with novel *MYORG* variants. Two nonsense variants (c.442C > T, p. Q148*; c.972C > A, p. Y324*) and two missense variants (c.1969G>C, p. G657R; c.2033C > G, p. P678R) of *MYORG* were identified in four sporadic PFBC patients, respectively. These four novel variants were absent in gnomAD, and their amino acid were highly conserved, suggesting these variants have a pathogenic impact. Patients with *MYORG* variants tend to display a homogeneous clinical spectrum, showing extensive brain calcification and parkinsonism, dysarthria, ataxia, or vertigo. Our findings supported the pathogenic role of *MYORG* variants in PFBC and identified two pathogenic variants (c.442C > T, c.972C > A), one likely pathogenic variant (c.2033C > G), and one variant of uncertain significance (c.1969G>C), further expanding the genetic and phenotypic spectrum of PFBC-*MYORG*.

## Introduction

Primary familial brain calcification (PFBC), widely known as Fahr’s disease, is a rare inherited neurodegenerative disease characterized by bilateral calcium deposits in the basal ganglia and/or other brain regions, in the absence of other secondary causes for brain calcification (Manyam, 2005). Affected individuals can exhibit a wide range of clinical symptoms, including dystonia, parkinsonism, ataxia, cognitive impairment, and psychiatric symptoms, while some remain asymptomatic for their entire lives (Nicolas et al., 2013a). The prevalence of PFBC is still unknown, and some studies indicate that it is underdiagnosed because of its unspecific presentations (Nicolas et al., 2018).

Typically, PFBC is inherited in an autosomal dominant manner (AD-PFBC); to date, four autosomal dominant PFBC-associated genes have been identified, including *SLC20A2*, *PDGFRB*, *PDGFB*, and *XPR1* (Wang et al., 2012; Nicolas et al., 2013b; Keller et al., 2013; Legati et al., 2015). Little is known about their mechanisms, which are thought to be related to phosphate homeostasis via mutations in *SLC20A2* and *XPR1*, and pericyte function affecting the blood–brain barrier integrity by mutations in *PDGFRB* and *PDGFB*. Our study demonstrated that *SLC20A2* accounted for the highest contribution (14.2%) in Chinese PFBC, followed by *PDGFRB*, *PDGFB*, and *XPR1* (0.9% each) (Guo et al., 2019). As such, a substantial proportion of patients remain genetically undiagnosed (Taglia et al., 2015). Recently, we have first reported disease-causing mutations in *MYORG* gene (MIM: 618255) for the autosomal recessive form of PFBC (AR-PFBC) (MIM #618317) (Yao et al., 2018). Another team identified *JAM2* as a gene related to autosomal recessive PFBC, further supporting the recessive pathogenic gene as a factor in PFBC pathogenesis (Cen et al., 2020).

There is currently little information about the mutational and clinical spectrum of Chinese patients with PFBC. In the present study, we analyze detailed genetic and clinical data from four PFBC patients carrying novel *MYORG* variants and investigate the relationship between mutations, phenotyping, and neuroimaging features.

## Material and Methods

### Subjects

A total of 245 patients with possible autosomal recessive traits or negative family history were recruited from multiple hospitals in China from January 2012 to August 2021, which had excluded the previously reported recessive families (Yao et al., 2018). The criteria for the diagnosis of PFBC were as follows: 1) bilateral and symmetrical calcifications in the basal ganglia and/or dentate nucleus detected by CT scans; 2) a total calcification score (TCS) rates above the age-specific thresholds (Nicolas et al., 2013a); 3) absence of biochemical abnormalities, including serum concentration of calcium, phosphate, and parathyroid hormone; 4) secondary causes of brain calcification were excluded such as infectious, toxic, or traumatic causes. All sporadic patients were previously tested for variants of all AD-PFBC genes by Sanger sequencing, and no pathogenic variants were detected (*SLC20A2*, *PDGFRB*, *PDGFB*, and *XPR1*). For all patients, a complete neurological examination was performed by two neurologists. The calcifications in the cerebral locations were further evaluated with TCS (range from 0 to 80). In cases with biallelic *MYORG* variants, previous investigations were retrospectively analyzed based on a chart review where available: neuroimaging in all reported cases with CT (*n* = 3) and brain MRI (*n* = 1). We also recruited 200 individuals without brain calcification as normal controls. The study was approved by the institutional review board at the First Affiliated Hospital of Fujian Medical University. All subjects provided informed consent before inclusion.

### Mutational Analysis

We collected venous blood samples from all participants including all available family members. Genomic DNA was extracted from peripheral blood leukocytes using standard protocols. Polymerase chain reaction (PCR) was performed to amplify the coding exon (exon 2) of the *MYORG* gene (NM_020702.5), using the primers previously reported (Yao et al., 2018). The PCR products were purified and analyzed by Sanger sequencing with ABI 3730XL automated DNA-sequencing system. The sequence data were further analyzed and compared with reference *MYORG* coding sequences from the Human Genome database (NM_020702.5).

The identified variants that fulfilled the following criteria were included for further analysis: 1) excluded variants with frequencies exceeding 0.1% in the 1,000 Genomes Project (www.1000genomes.org), Exome Aggregation Consortium (ExAC, http://exac.broadinstitute.org/), or Genome Aggregation Database (gnomAD v2.1.1; http://gnomad.broadinstitute.org); 2) excluded synonymous variants and missense variants, which were predicted to be nonpathogenic *in silico* by Mutation Taster (http://www.mutationtaster.org/), Polyphen2 (http://genetics.bwh.harvard.edu/pph2/), or SIFT (http://sift.jcvi.org/). The evolutionary conservation of the affected amino acid among different species was estimated using HomoloGene (http://www.ncbi.nlm.nih.gov/homologene). All novel variants were independently classified by two investigators based on the American College of Medical Genetics and Genomics (ACMG) guidelines and the Association for Molecular Pathology (Richards et al., 2015).

## Results

### *MYORG* Variants in the Primary Familial Brain Calcification Cohort

**FIGURE 1:**
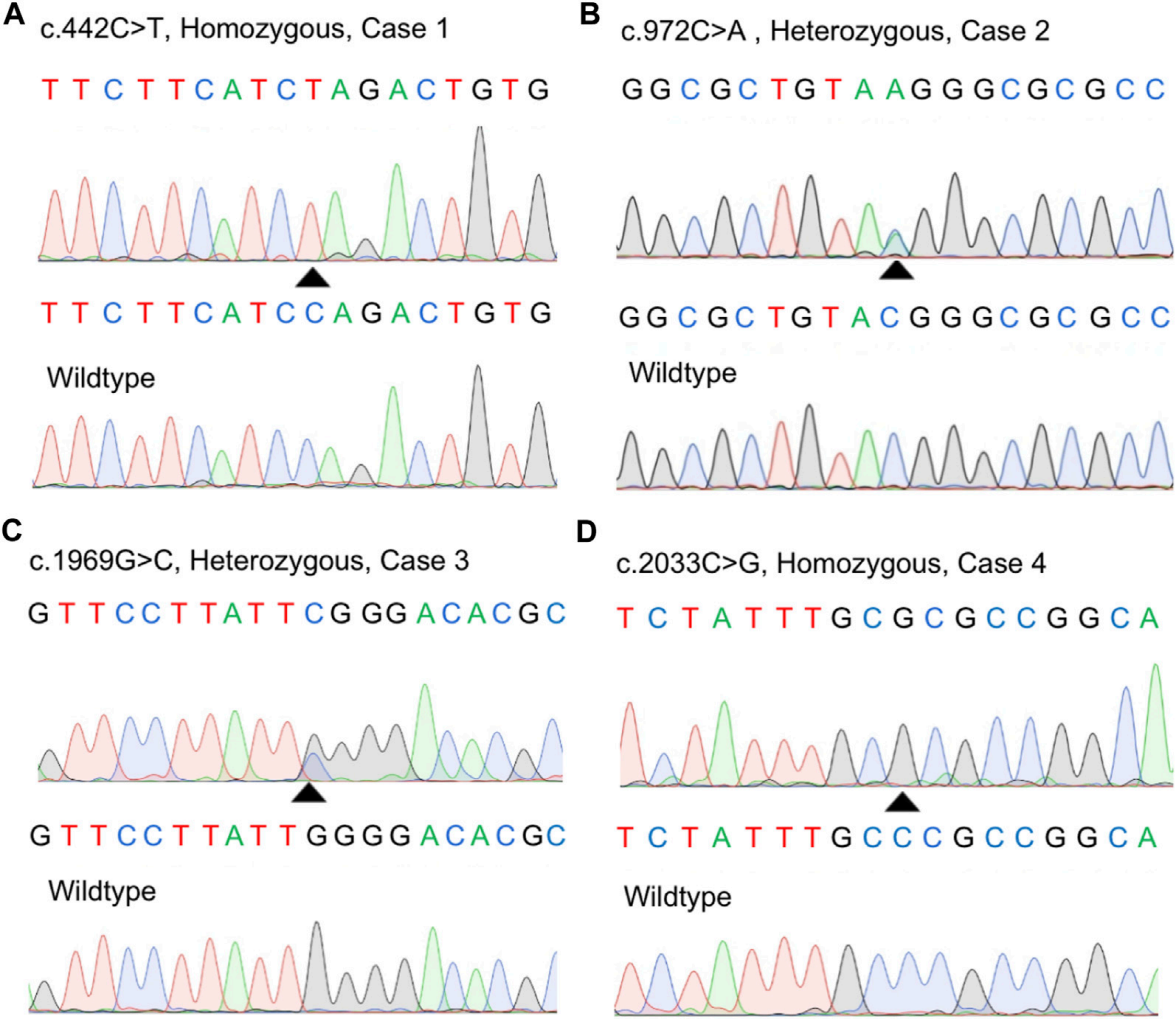
Identification of four novel variants in our study. **(A–D)** Sanger sequences of the *MYORG* variants in the four cases and corresponding wild-type subjects.

We screened a total of 245 Chinese subjects including 21 subjects from 10 possibly autosomal recessive families and 224 sporadic cases. Eight *MYORG* variants were identified in six sporadic cases, while four of the eight variants were previously reported (c.103A > G, p.M35V; c.782_783GC > TT, p. R261L; c.1092_1097delCTTCGA, p.365_366delFD; and c.1967T > C, p. I656T) (Supplementary Figures S1A–D) (Yao et al., 2018; Forouhideh et al., 2019; Chelban et al., 2020; Chen et al., 2020). Notably, four novel variants in *MYORG* were identified: two nonsense variants (c.442C > T, p. Q148*; c.972C > A, p. Y324*) and two missense variants (c.1969G>C, p. G657R; c.2033C > G, p. P678R) (Figures 1A–D). The variants, c.442C > T and c.2033C > G, were separately found in a homozygous state in case 1 and case 4, while c.972C > A and c.1969G>C were identified in a heterozygous state in case 2 and case 3, respectively coupled with the previously reported variants (c.103A > G and c.782_783GC > TT) (Table 1).

**TABLE 1:** Clinical features in PFBC patients with *MYORG* mutations F, female; M, male; AAE, age at examination; AAO, age at onset; y, years; TCS, total calcification score; NA, not available.

Case No	Sex	AAE (y)	AAO (y)	Clinial features	Calcification		Zygosity	cDNA alteration	Amino acid alteration	GnomAD frequency (global)	Mutation Taster	SIFT	PolyPhen -2	CADD	ACMG Classification
					Localization	TSC									
1	M	72	66	dysarthria, parkinsonism, vertigo	globus pallidus, thalamus, dentate nuclei, subcortical white matter	18	homozygous	c.442C > T	p.Q148*	absent	Disease causing	NA	NA	36.0	Pathogenic
															PVS1+PM2+PM3+PP3
2	F	47	42	dysarthria, dysphagia, forced laughter or crying	basal ganglia, thalamus, dentate nuclei, subcortical white matter	43	heterozygous	c.972C > A	p.Y324*	absent	Disease causing	NA	NA	34.0	Pathogenic
															PVS1+PM2+PP3
								c.103A > G	p.M35V	0.000289	Disease causing	Tolerated	Probably damaging	24.0	Likely pathogenic
															PS1+PM2+PP3+PP5
3	F	68	45	vertigo	basal ganglia, dentate nuclei, thalamus, corona radiata	NA	heterozygous	c.1969G>C	p.G657R	absent	Disease causing	Damaging	Probably damaging	28.9	Variants of uncertain significance
															PM1+PM2+PP3
								c.782_783 GC>TT	p.R261L	absent	Disease causing	Tolerated	Benign	NA	Likely pathogenic
															PS1+PM2+PP5
4	M	62	59	dysarthria, parkinsonism, ataxia	basal ganglia, thalamus, dentate nuclei, subcortical white matter, cerebellar vermis, brainstem	57	homozygous	c.2033C > G	p.P678R	absent	Disease causing	Damaging	Probably damaging	28.3	Likely pathogenic
															PM1+PM2+PM3+
															PP3+PP4

### Evaluation of the Pathogenicity of the Three *MYORG* Missense Variants

**FIGURE 2:**
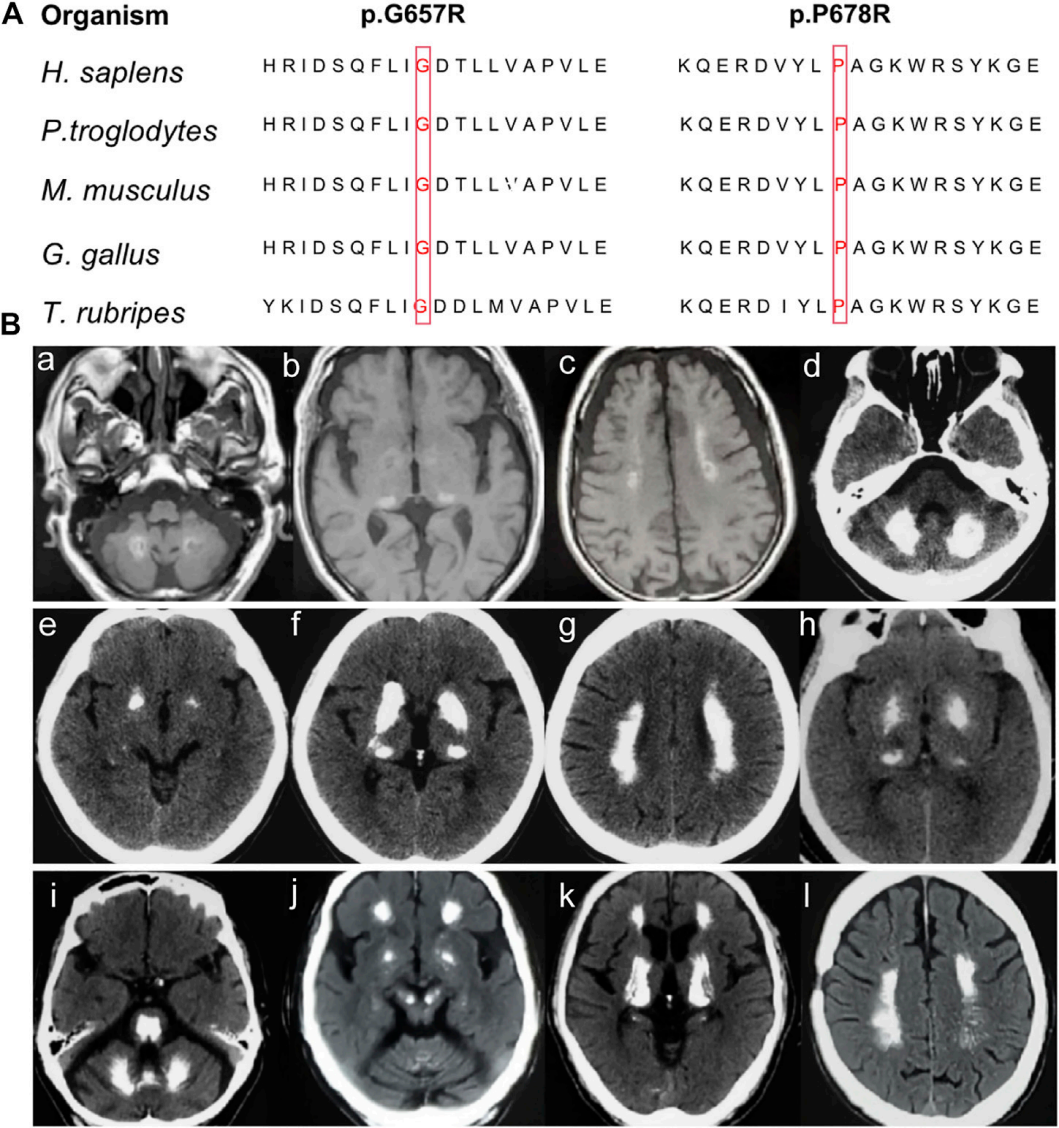
**(A)** Conservation analysis across species for the novel missense *MYORG* variants. The variants were marked with red boxes for the corresponding amino acid. **(B)** Neuroimaging spectrum in the four cases. Cerebral MRI of Case 1 **(Ba–c)** showed high intensity in the globus pallidus, thalamus, dentate nuclei, and subcortical white matter; CT scan of case 2 **(Bd–g)** showed symmetrical calcification at the basal ganglia, thalamus, dentate nuclei, and subcortical white matter; case 3 **(Bh)** showed calcification in the basal ganglia and thalamus; and case 4 **(Bi–l)** showed prominent calcification in the basal ganglia, thalamus, dentate nuclei, cerebellar vermis, subcortical white matter, and brainstem.

None of the novel variants mentioned above were present in the 1000G, ExAC, or gnomAD (Table 1). *In silico* analysis predicted deleterious consequences using the Mutation Taster, SIFT, Polyphen2, and CADD software programs. The two missense variants were both located at the highly conserved positions and the glycosidase domain (Figure 2A). Based on the American College of Medical Genetics (ACMG) guidelines, the two nonsense variants were considered as “Pathogenic;” the missense variant c.2033C > G was considered as “Likely Pathogenic;” and c.1969G>C was considered as “Variants of Uncertain Significance” (Table 1). Four of the 224 sporadic PFBC (1.79%) carried variants in *MYORG* in this study.

### Clinical Manifestations of the Patients Carrying Novel *MYORG* Mutations

The clinical manifestations of the patients with novel *MYORG* variants are summarized in Table 1. All four patients showed extensive calcification involving the basal ganglia, dentate nuclei, thalamus, and the subcortex in CT/MRI scans. Patients displayed a homogeneous clinical pattern and commonly experienced parkinsonism, dysarthria, ataxia, and vertigo. Detailed clinical and radiological findings of the patients are described as follows.

Case 1. The patient with a c.442C > T nonsense homozygous variant was a 73-year-old man who was admitted to the hospital because of dysarthria, gait impairment, and vertigo. These symptoms were experienced for 7 years. There was an initial progressive slurred speech, walked unsteadily, starting hesitantly with reduced up and down gaze, associated with profound bilateral bradykinesia, and a combination of ataxia and freezing. Progressive deterioration of dysarthria and bradykinesia became evident over the following years. He had difficulty in tandem walking and could not walk independently. A neurological examination revealed cerebellar dysarthria, supranuclear gaze palsy, bradykinesia, festinating gait, and mild rigidity in the lower limbs; no tremor was observed. His cerebral MRI at age 72 years showed high-intensity in the globus pallidus, thalamus, dentate nuclei, and subcortical white matter, suggesting moderate symmetrical calcification (Figures 2Ba–c). The patient reported no family history of brain calcification.

Case 2. The patient, carrying c.972C > A and c.103A > G heterozygous variants of *MYORG*, was a 47-year-old woman who had slurred speech with slow progression. She later developed dysphagia and forced laughter or crying. No other abnormalities were observed, including gait disorder, bradykinesia, involuntary movements, or psychiatric disorders. A neurological examination revealed dysarthria, forced laughter, and a positive Huffman’s sign. Her CT images (TCS, 43) showed severe calcification at the bilateral basal ganglia, thalamus, dentate nuclei, and subcortical white matter (Figures 2Bd–g). Unfortunately, her parents were unable to provide brain CT or blood samples.

Case 3. The patient who carried c.1969G>C and c.782_783GC > TT heterozygous variants was a 68-year-old female from Northern China. She presented with 23 years of vertigo and had a previous history of cerebral infarction, cerebral hemorrhage, hypertension, and coronary heart disease. A neurological examination was unremarkable. Laboratory tests showed normal levels of serum calcium, phosphorus, and parathyroid hormone. Her brain CT reported symmetrical calcification at the basal ganglia, thalamus, dentate nucleus, and corona radiata though a complete series of images was not available (Figure 2Bh). Unfortunately, we could not examine her parents because they have both passed away.

Case 4. The 62-year-old man carrying the homozygous c.2033C > G variant experienced disease onset at the age of 59, with slowly progressive gait unsteadiness, reduced up and down gaze and slurred speech. A neurological examination revealed mild dysarthria, bradykinesia, rigidity of the limbs, staggering in tandem gait, and poor pointing performance of the finger-to-nose test. No other abnormalities were found, including psychiatric disorder, seizure, memory disturbance, or involuntary movements. His CT images (TCS, 57) revealed prominent calcification in the basal ganglia, thalamus, dentate nuclei, cerebellar vermis, and subcortical white matter (Figures 2Bi–l). Extensive brainstem calcifications affecting the pons and mesencephalon were also observed. Moreover, cerebellar atrophy could also be witnessed in the CT images (Figures 2Bi-j). Unfortunately, brain CT and genetic screening of his parents were unavailable due to their death.

## Discussion

Our previous study suggests that mutations of *MYORG* are the main cause of autosomal recessive PFBC (Yao et al., 2018). To date, more than 50 variants associated with PFBC have been reported in different ethnic populations, further confirming the pathogenicity of *MYORG* mutations (Bauer et al., 2019; Westenberger et al., 2019). In this study, we identified four novel variants of *MYORG* including two missense variants and two nonsense variants in four sporadic cases. All of these variants were absent in any control individuals indicating that they were likely to be deleterious variants. The variants c.442C > T and c.972C > A both led to the premature termination codon (p.Q148* and p. Y324*) and resulted in a truncated protein. The two missense variants, c.1969G>C, p. G657R and c.2033C > G, p. P678R, were both located in glycosyl hydrolase domain (aa 311–714) and the highly conserved regions, resulting in an amino acid substitution that was predicted to negatively affect protein function. According to the standards and guidelines for the interpretation of sequence variants, c.442C > T and c.972C > A could be considered as pathogenic variants with the evidence of PVS1 + PM2 + PM3 + PP3 and PVS1 + PM2 + PP3. Also, c.2033C > G could be considered as a likely pathogenic variant based on existing evidence of PM1 + PM2 + PM3 + PP3 + PP4. However, we could not confirm that it fits an autosomal recessive pattern due to lack of examination of the parents of case 2. Therefore, we classified this variant c.1969G>C as “uncertain significance” at this time point based on the evidence of PM1 + PM2 + PP3.

The role of the MYORG protein (known as NET37 or KIAA1161) is largely unknown. Based on sequence analysis, MYORG is predicted to be a member of the glycosyl hydrolase 31 family, with a glycosidase function (Datta et al., 2009). Our previous study suggested that MYORG was specifically expressed in astrocytes, a key component of the neurovascular unit (NVU), and mutations of *MYORG* would result in dysfunction in NVU. MYORG protein contains a transmembrane domain at its N-terminus and a family 31 glycosyl hydrolase domain at its C-terminus. The two missense variants in our patients were both located in the glycosidase domain, and the two nonsense variants would result in a truncated protein without glycosidase domain, suggesting that the region plays an important role. The four novel variants in our patients could induce loss of glycosidase activity and, thus, lead to PFBC. However, additional study is needed to clarify the relationship between this mutations and resulting protein functions.

The clinical manifestations and neuroimaging features significantly vary among PFBC patients with mutations in different pathogenic genes. In our four patients, the brain CT findings showed widespread and abundant calcifications, which is consistent with the neuroimaging features in PFBC patients with *MYORG* mutations as reported by Chen et al. (Chen et al., 2019; Chen et al., 2020). Compared with other pathogenic genes, brainstem calcifications affecting the pons and cerebellar atrophy could be the prominent features in *MYORG* mutation carriers. This seems to be an indicator of this genetic form (Grangeon et al., 2019; Kume et al., 2020). Extensive calcifications encompassing the pons and extending to the whole brainstem were observed in case 4. However, the calcifications in the basal ganglia and the cerebellum could not be distinguished from individuals with mutations of other PFBC pathogenic genes.

Patients with *MYORG* mutations tend to display a homogeneous clinical spectrum, showing dysarthria, parkinsonism, gait disorder, and ataxia (Taglia et al., 2019). Our patients also displayed dysarthria and parkinsonism as the major symptoms. As reported, dysarthria was shown to be a common prominent feature in the majority of symptomatic cases with *MYORG* mutations compared with those with mutations in the dominant-causing genes (Ramos et al., 2019). Parkinsonism was another common feature in our patients with *MYORG* mutations shown in case 1 and case 4. Parkinsonism with vertical nuclear gaze palsy was uncommon in *MYORG* mutation carriers, but occurred in case 4, thus, extending the phenotypic spectrum of *MYORG*-related PFBC. Parkinsonism with vertical nuclear gaze palsy was reported to be associated with pontine calcifications, which were also noticed in the brain CT of case 4 (Chelban et al., 2020). In our study, homozygous or truncating mutations of *MYORG* could be associated with a more severe phenotype, such as in case 1 and case 4.

It is possible that we underdiagnosed the *MYORG* mutations in our PFBC cohort due to certain limitations. First, we did not detect duplications or deletions of the total or part of the *MYORG* gene in the cohort. We also could not recruit familial members of the patients with *MYORG* variants for the co-segregation analyses. However, some *MYORG* synonymous variants found in this study could influence splicing.

The clinical symptoms and neuroimaging characteristics identified by this study, could aid clinicians in orienting genetic testing for PFBC, combining with the recessive inheritance patterns. In conclusion, we reported four novel *MYORG* mutations in Chinese PFBC, expanding the genetic and phenotypic spectrum of this disease.

## Data Availability

All of the data supporting the findings in this study are available upon reasonable request from the corresponding authors.

## Supplementary Material

Supplementary Figure S1 Identification of four previously reported variants in our study. **(A–D)** Sanger sequences of the previously reported variants and corresponding wildtype subjects.
