# Supplementary figures and images for: Mutation Analysis of MYORG in a Chinese Cohort With Primary Familial Brain Calcification
Source: Front Genet. 2021 Oct 18;12:732389. doi: 10.3389/fgene.2021.732389 (PMC8570371; doi:10.3389/fgene.2021.732389)

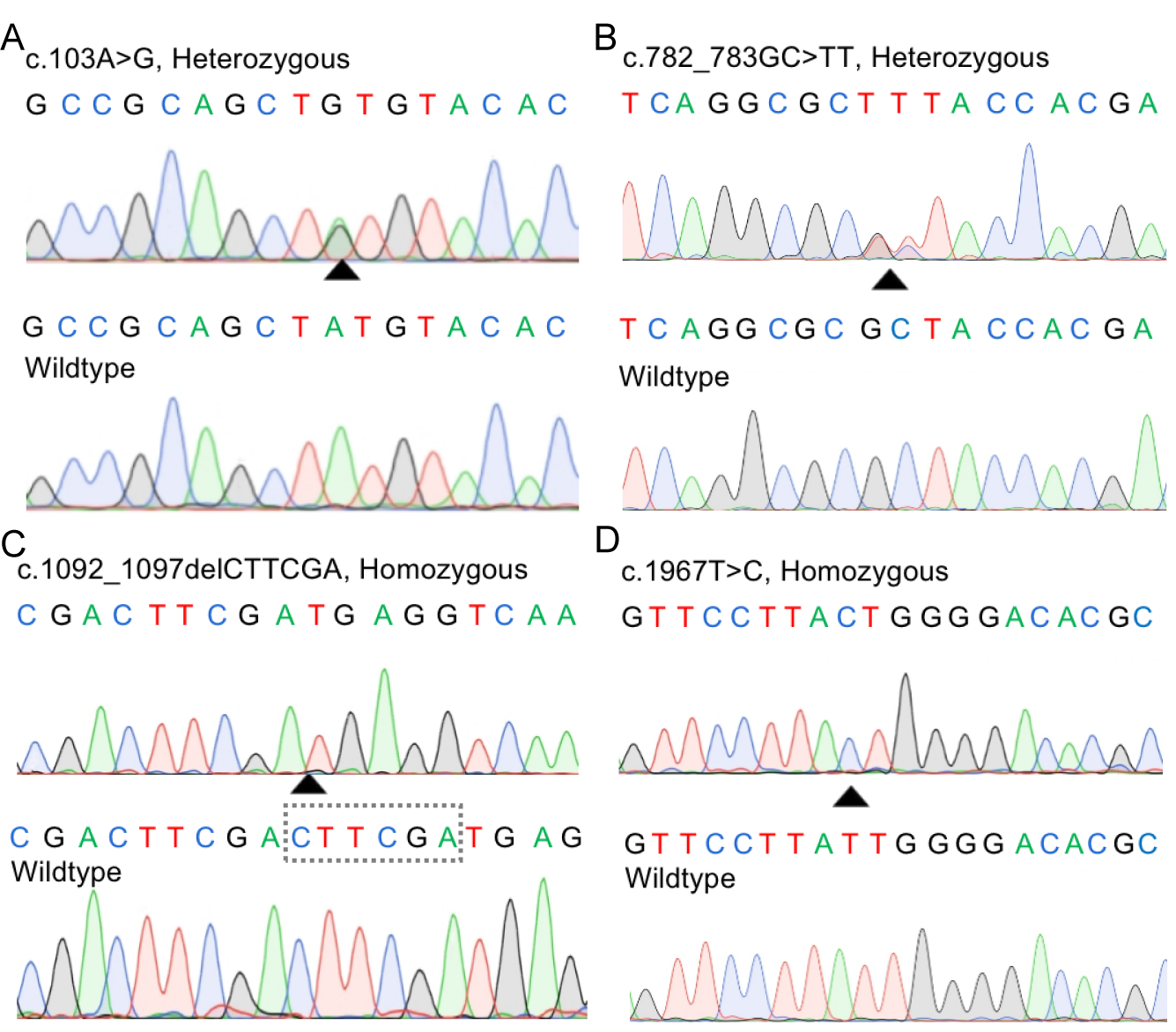

Supplement: Supplementary file 1 [file Image1.tif]
